# Description of complex interventions: analysis of changes in reporting in randomised trials since 2002

**DOI:** 10.1186/s13063-018-2503-0

**Published:** 2018-02-22

**Authors:** Bridget Candy, Victoria Vickerstaff, Louise Jones, Michael King

**Affiliations:** 1 Marie Curie Palliative Care Research Department, Division of Psychiatry, University College London, 6th Floor, Maple House, 149 Tottenham Court Road, London, W1T 7NF UK; 2 Division of Psychiatry, University College London, 6th Floor, Maple House, 149 Tottenham Court Road, London, W1T 7NF UK

**Keywords:** Trials, Reporting standards, Complex intervention content, CONSORT, TIDieR

## Abstract

**Background:**

Inadequate description of non-pharmacological complex interventions in trial publications means that they cannot be replicated or assessed for generalisability. There are published guidelines on how to describe an intervention, such as those from the CONSORT Group. However, there have been few evaluations of whether intervention reporting is improving.

**Methods:**

We aimed to assess whether descriptions of multicomponent, non-pharmacological interventions evaluated in randomised trials are improving. To do so, we chose trials of educational and psychotherapeutic interventions to promote adherence to therapy, and compared those published between 2002 and 2007 (Time-1) with those between 2010 and 2015 (Time-2). These time periods were chosen to concord with the publication in 2008 of the CONSORT extension statement of reporting guidelines for non-pharmacological treatment which included items on intervention description. We assessed 19 items, based on the CONSORT Statement and the more recent Template for Intervention Description and Replication Checklist (TIDieR). Two reviewers independently extracted data. We created a quality score of the eight items we considered key information for replication and assessment of generalisability (setting, provider, recipient, comparator, intervention intensity, how it was conducted, existence of a manual or protocol, and detail of whether there was an assessment of fidelity). Score per item was ‘1’ if reported adequately and ‘0’ if not.

**Results:**

Of the eligible trials, 42 were published in Time-1 and 134 published in Time-2. The trials included were published in 112 peer-reviewed journals, 52 of these journals currently require authors to follow the CONSORT Statements, while only one recommended adherence to the TIDieR.

Most items of CONSORT and TIDieR were reported by more than half of the trials at both time points. Few trials reported fidelity. A large proportion of the trials did not report the existence of a manual or protocol, or what the comparator group received. We found no statistically significant improvement in the eight-item quality score (Time-1: mean 5.71 (standard deviation (SD) 1.09), Time-2: 5.87 (SD 1.28), *p* = 0.49).

**Conclusions:**

We found no overall evidence that reporting the specifics of multicomponent, non-pharmacological interventions is improving. Details to replicate interventions remain lacking, impairing best implementation or meaningful further research. Editorial endorsement of reporting checklists needs to be more extensive.

## Background

The development of effective healthcare interventions relies on appropriate design and evaluation. There are guidelines to assist in intervention development, including from the Medical Research Council (MRC) on complex interventions [1]. The MRC guidance recommends a careful process of development of underlying theory, modelling of process and outcomes, followed by assessment of feasibility and eventual dissemination. To enable dissemination, adequate description of any intervention is required so that it can be replicated and tested in other populations and settings, or generalised and applied across contexts [2, 3]. Limitations in descriptions of interventions reported in publications in which these interventions have been tested in randomised trials may waste resources and potentially harm patients [4].

Reporting of complex, non-pharmacological interventions may be challenging. Such interventions may be multi-faceted; there may be several components and it may be unclear which of these components provide the ‘active ingredient(s)’ [1]. Moreover, both the effectiveness and the replicability of a non-pharmacological intervention may be reliant not only on how it is provided, but by whom [5]. Fidelity to protocol in the conduct of the intervention may be challenging, in part as an intervention may need to be tailored to the recipients [6]. However, testing of complex interventions in randomised controlled trials is needed with clear descriptions of the content of the intervention and its procedures [7].

Recognition of the need to provide clear and complete reporting is not new. The Consolidated Standards of Reporting Trials (CONSORT) guidance first published in 1996 aims to improve trial reporting [8]. Consort reporting statements are held in high regard. Peer-reviewed journals and editorial publishing groups endorse them, for instance by providing direct links to CONSORT websites and requiring their use in submitted manuscripts of clinical trials. The 2008 extension to CONSORT Statements for non-pharmacological treatment [9] takes into special consideration the issues in reporting of these treatments, including intervention complexity and delivery (Table 1). A more recent development that aims to enhance intervention reporting further is the Template for Intervention Description and Replication (TIDieR) Checklist [10]. This checklist includes 12 domains. The endorsement of TIDieR has yet to be taken up as extensively by journals as the CONSORT. Both TIDieR and CONSORT are listed for use in the international publishing and writing initiative to Enhancing the QUAlity and Transparency Of health Research (EQUATOR) network [11].

**Table 1:** The Consolidated Standards of Reporting Trials (CONSORT) extension statement for non-pharmacological treatments: items specific to intervention description and its implementation

When applicable, eligibility criteria for centres and those performing the interventions	
Precise details of both the experimental treatment and comparator	
Description of the different components of the interventions and, when applicable, descriptions of the procedure for tailoring the interventions to individual participants	
Details of how the interventions were standardized	
Details of how adherence of care providers with the protocol was assessed or enhanced	

There are, to our knowledge, limited comparative evaluations on whether reporting of interventions is improving, none have compared two recent 5-year periods separated by publication of a new reporting guideline. In this paper, we explored whether the quality of the descriptions of non-pharmacological interventions has improved by comparing trials published in the 5 years prior to the 2008 publication of the CONSORT extension statement for non-pharmacological treatments with those published between 2010 and 2015.

## Methods

We used, as an example, published papers reporting trials of interventions to promote adherence to therapy. We selected these type of interventions as we were aware of multiple trial evaluations, and because the interventions are varied, complex and multicomponent.

### Data source

**Fig. 1:**
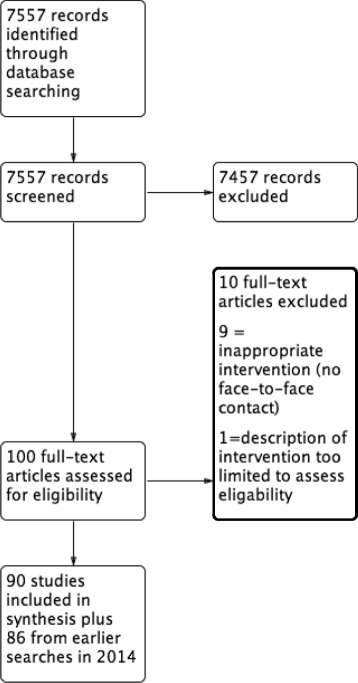
Review flow chart

We used trials included in the latest version of a Cochrane review on interventions for enhancing medication therapy adherence [12], but updated their search from 2013 to 2015. To do this we re-ran their search terms (see Appendix) in the same six citation databases they used (namely: CINAHL (via EBSCO), MEDLINE, EMBASE and PsycINFO (all via Ovid), Sociological Abstracts (via ProQuest) and Cochrane CENTRAL). We applied their inclusion criteria to identify relevant trials of these interventions. See review flow chart, Fig. 1.

### Our inclusion criteria

We included peer-reviewed journal publications of the main findings from randomised controlled trials of psychotherapeutic and/or educational interventions to promote adherence to therapy. These trials were selected because the interventions are varied in their components and how they are provided. These complex variations highlight the need for precise reporting of the intervention.

We included only those papers which evaluated a multicomponent intervention and for which there was some detailed description (more than one sentence) concerning the intervention content. This restriction allowed a more useful application of intervention-reporting guidelines. We also only included those interventions that involved face-to-face contact as opposed to those, for example, where the intervention was provided online or via telephone contact. This restriction aimed to ensure greater similarity in aspects of the intervention for which it was reasonable to expect a clear and comparable description to have been included in the report of the trial (e.g. who delivered the intervention).

Trials were only included if they were published within two 5-year time periods; 2002 to 2007 (Time-1) and 2010 to 2015 (Time-2). We chose a gap of several years between the time periods to allow time for any impact of the 2008 extension to CONSORT Statements for non-pharmacological treatment on trial reporting of interventions [9].

### Outcomes of interest

Our primary outcome of interest was the quality of the description of the intervention in terms of its:

- Potential for replicability in clinical practice or in subsequent trials (e.g. content, intensity and how delivered)
- Assessment for generalisability (e.g. setting, recipient and provider)

Secondary outcomes included:

- Items that provide a greater understanding of the development of the intervention, such as theory to underpin the intervention or whether patients’ views had informed development
- Items that may provide additional description of the trial such as supplementary materials or descriptive aids such as a table or figure, and even the trial’s title
- Items that may be more important to describe for some trials such as where the intervention is shaped or fitted to the requirements of each individual
- Items on other ways that may measure improvement in reporting such as the word length devoted to intervention description
- Whether there are differences in reporting between trials published in journals that currently require trials to following CONSORT Statements or other reporting guidelines, and those that do not
- Whether there are differences between trials published in journals with higher compared to lower impact factors

### Data handling and analysis

We extracted data guided by 19 categories/items relating to the development of the intervention, the description of the intervention and the description of any comparator (control or another intervention). These items are detailed in Table 2. They are based on the CONSORT extension statement [9] and the TIDieR Checklist [10], although most items are derived from the more extensive TIDieR Checklist. We extracted data on two additional items: the length of text devoted to the description of the intervention and the comparator; and whether the patients’ views were considered in the development of the intervention. We also noted how many of our included trials were published in journals that now recommend the use of the CONSORT and TIDieR Statements and the current impact factor of the journal.

**Table 2:** Description of items extracted

Item label	Description of item reported in the publications
Brief name	Precise name or phrase describing the intervention
Rationale	Rationale, theory or goal for testing the intervention components
Patient views’	Patient feedback incorporated in intervention development
Setting	Where the intervention was conducted
Recipient	Inclusion criteria provided for the recipients
Provider	Sufficient details on who provided the intervention. For example, type of professional or volunteer, their expertise and background and whether they received any training to undertake the intervention
How	Description of the procedures, activities, and/or processes used in the intervention, including any enabling or support activities
Tailored	Intervention tailored to the recipient, i.e. did it consider the participants’ circumstances or wishes in regards to how they adhered to medication?
Manual or protocol	Intervention has been standardized such as in a manual, procedure book or protocol
Intervention sessions	Number of intervention sessions provided
Duration of intervention	Length of time over which the intervention provided was given
Duration of sessions	Indication of the length of each session
Modifications	Modification of the intervention during the study
Intervention supporting materials	Description of supporting materials, including those provided to participants or used in intervention delivery or in training of the intervention providers
Descriptive aids	Details of the intervention provided in the paper in a table, figure or another medium
Supplementary materials	References or electronic links to additional information describing the intervention
Comparator arm	Any details on what participants in the comparator group received
Fidelity	Was the conduct of the intervention observed or recorded? If so: • How was this done? • Who assessed fidelity? • How did the study seek to maintain fidelity? • Did they report how well fidelity was maintained?
Word length	Length of text devoted to describing the intervention and comparator.

Two authors independently extracted the data (BC, VV). Each item was coded on whether it was adequately reported, scoring ‘1’ if yes or ‘0’ if not*.* Our individual assessments for all items were compared and any differences discussed between us. Should consensus not have been reached, we would have referred to discussion with the other authors (LJ, MK).

Not all the data that we extracted were relevant to our main outcome of interest (the provision of sufficient information for replication and assessment of generalisability of outcomes). To enable us to answer our primary aim we created a total quality score of the eight items that we considered provided information needed for replication and generalisability. The items concerned description of the:

- Setting
- Intervention provider
- Recipient
- Comparator arm

and documentation of:

- The number of sessions the intervention involved
- How the intervention was provided
- A manual or protocol
- Whether fidelity of conduct of the intervention was assessed

We totalled the score for these items per time point.

Data were summarised using descriptive statistics. T tests were used to compare quality scores between the time periods and between those which were published in a journal that requires the use of a CONSORT Statement or other reporting guidelines and those which do not. Correlations were undertaken between the journal impact factor and overall quality score. Statistical analysis was performed using Stata software version 13.

## Results

Forty-two trials published in Time-1 and 134 published in Time-2 were eligible for inclusion (See Fig. 1). Table 3 presents how the items were reported. For many items reporting in both time periods was similar. In Time-1, eight items were adequately reported and in Time-2 six items were adequately reported in 90% or more of trials; for both time periods these included provision of a rationale for the intervention, inclusion criteria for the recipient of the intervention and detail on how the intervention was provided. Some items were consistently under-reported in a large proportion of studies, in particular, fidelity was reported as being assessed in 24% of the trials at Time-1 and in 28% at Time-2. There was no description of the comparator in 40% of studies at Time-1 and in 34% at Time-2. Very few trials, 5% at both times, reported that patients’ views were considered in the development of the intervention. Some items were reported more often in Time-2: these were rationale, the existence of a protocol or manual, the existence of supplementary materials and whether or not the intervention had been tailored. Setting was more often reported in Time-1.

**Table 3:** Number of items reported (*n* (%))^a^ grouped by 5-year time periods

	Time-1 2002–2007 (*n* = 42)	Time-2 2010–2015 (*n* = 134)
Intervention name	42 (100)	120 (90)
Rationale for trial	39 (93)	132 (99)
Patient’s views	2 (5)	7 (5)
Setting	40 (95)	98 (73)
Recipient	41 (98)	132 (99)
Provider	37 (88)	120 (90)
Manual or protocol	12 (29)	75 (56)
Intervention sessions	35 (83)	115 (86)
Duration of intervention	33 (79)	110 (82)
Duration of individual sessions	17 (40)	74 (55)
How	40 (95)	120 (90)
Tailored	17 (40)	78 (58)
Participant supporting materials	15 (36)	62 (46)
Fidelity assessed	10 (24)	37 (28)
How fidelity assessed	10 (100)	36 (95)
Who assessed fidelity	8 (80)	28 (74)
Fidelity outcome	10 (100)	13 (34)
Adherence to intervention	3 (30)	12 (32)
Supplementary materials	5 (12)	34 (25)
Descriptive aids	9 (21)	35 (26)
Modifications	1 (2)	4 (3)
Comparator	25 (60)	88 (66)
Number of words (mean, SD)	436.7 (SD 272)	406.1 (SD 338.3)
Number of sentences (mean, SD)	17.7 (SD 11.7)	17.1 (SD 10.0)

^a^Including fidelity sub-items. See Table 2 for further details on items. *SD* standard deviation

Using our overall quality score, we found no statistically significant improvement in reporting of the eight items we considered key information for replication and assessment of generalisability of these interventions (Time-1: mean 5.71 (standard deviation (SD) 1.09), Time-2: 5.87 (SD 1.28), *p* = 0.49.

Trials were published in 112 different peer-reviewed journals; of these journals 52 currently (November 2017) require authors to follow the CONSORT Statements, one specified  the use of the TIDieR guidance. There was no statistically significant difference in reporting quality between trials published in journals that currently recommend CONSORT or other reporting guidelines (such as TIDieR) and those that do not. The average quality score for those which do not have reporting requirements was 5.75 (5.49, 6.01) and for those which do have reporting requirements was 5.99 (5.73, 6.24) (mean difference = − 0.240, 95% confidence interval(CI) − 0.61, 0.13, *p* = 0.20). There was no statistically significant association between the reporting quality of the trials and the current impact factor of the journals in which they were published (coefficent = 0.02, (95% CI − 0.01, 0.05)), *p* = 0.15.

## Discussion

### Summary of key findings

We assessed whether descriptions of complex interventions in randomised trials are improving. To do so, we chose trials which evaluated multicomponent educational and psychotherapeutic interventions to promote adherence to therapy. We found no overall evidence that reporting of intervention content is improving. Descriptions in trial papers remained poor, in particular, whether a protocol or manual describing the intervention existed and any details on what the comparator intervention or control involved. One reason that reporting guidelines on intervention description have had little impact may be because a notable proportion of peer-reviewed journals do not currently require trial authors to use  reporting guidelines. The TIDieR guidance supersedes the CONSORT extension statement in regards to reporting the intervention. It includes a clearer understanding of what detail may be included when reporting a trial of an intervention; however, only one of the journals where papers in our sample were published currently recommends its use.

### Outcomes assessed

We sought to evaluate whether the reporting of interventions had improved as this is key to better assessments of the generalisabilty of the intervention and to replicate it. However, we recognize the challenges in our evaluation. Some items that we assessed concerned aspects of development and design, while others concerned the quality of how the trial was conducted. Some items overlapped, for instance one considered access to a manual and another access to supplementary material; both types of documents may hold similar details. Other items may have been more relevant for some trials than others. Our team held several discussions on what items to include in an overall quality score (on an adequate description for replication and assessment of generalisability). We are aware that our final selection may fit some of the included trials better than others.

We are also aware that more detail on the interventions may have been found in supplementary materials, such as a published protocol. Our focus, however, was on what could be gleaned from the main trial papers. Importantly, some of the items missing in substantial proportions of studies would not have taken many additional words to describe. Therefore, word limitations would not necessarily be a reason for their exclusion. More journals are provided in e-format only and thereby word restrictions are becoming less of an issue. Even reading a protocol will not necessarily provide information on whether all components planned were implemented as described. Since a substantial proportion of studies at both time points did not report the existence of other information, we believe that our evaluation is meaningful.

### Uptake of guidelines and journal publication

We did not restrict our focus to journals that recommend the CONSORT Statements or use other guidelines on reporting the intervention. However, since the CONSORT guidance is a well-cited tool, we assumed that these guidelines would have been taken up by authors and peer reviewers of their manuscripts. Our choice of not restricting by journal may be viewed as a limitation. Our lack of restriction however, highlights the limited endorsement of reporting guidelines amongst journals. We compared CONSORT and other guideline endorsers with non-endorsers. While we found no statistical significant difference in reporting quality between them, our analysis was based on whether the journal *currently* endorses reporting guidelines and not whether it did so in the year of publication of each trial. Therefore, our analysis may have under-estimated the impact of reporting guidelines as some trials will have been published some years prior to this requirement.

We found no association between the quality of reporting of the intervention in trials and the current impact factor of the journals in which they were published. We are aware that current impact factors may not reflect that at the time each paper was published. However, while such factors change, the relative rankings often do not.

The TIDieR guidance provides more detail on what to consider when describing an intervention and we found most items of the guidance easy to apply to our data as they were clearly expressed. However, some items remained open to differences in interpretation.

### Why have we found no improvement?

Trials continue to under-report intervention descriptions. The reasons for this are multiple. There remains a lack of awareness of the need for adequate reporting as many journals still do not endorse the use of reporting guidelines. However, even when they do it is likely that these policies are not always closely adhered to. Authors may also have little steer from journals on what to include in regards to intervention description, even from those that endorse the CONSORT guidelines. The items on intervention description in the CONSORT extension statement for non-pharmacological treatments are limited. Important details, such as the length and intensity of the intervention, are not specified (see Table 1). While the more recent TIDieR guideline provides more direction it was only endorsed in one journal in this cohort. Another reason for under-reporting is that some authors, novice or experienced, may feel overwhelmed by the growing number of reporting tools and may not know which to select [13]. Furthermore, authors may be challenged because of the complexity of the intervention and their own lack of clarity on the key details (or active ingredients) that are essential to report. They may, for example, fear over-simplifying in providing a summary of the intervention’s content and delivery. Should this arise there is in general the opportunity to publish or make available the intervention on request in the format of a protocol, manual or separate paper describing the intervention in detail. However, this does not seem to be happening. In the more recent cohort in our study only just over half (56%) of the authors documented a link to further intervention details.

Protocols are still not being published. Reasons may include authors’ lack of awareness of the opportunity to publish a protocol. Some may not appreciate the value of devoting additional time to this endeavour. Not all journals may accept protocols. Some authors may not want to publish a protocol in case the intervention deviates in testing from what was originally set out in the protocol. While changes to intervention content are acceptable and should be documented, some authors may be unaware of this. Others may be aware but have limited time to devote to justifying this in trial publications.

### Poor reporting is a wider issue

Our finding that descriptions of complex, non-pharmacological interventions in randomised trials has not improved is perhaps no surprise. There are previous reviews that have assessed changes in the quality of reporting of trials (e.g. [14–18]). In those concerning the reporting of the trial methods, improvements have been found [14–16]. For example, one study evaluated reporting over three decades of 20,920 randomised controlled trials and found improvement in reporting of six methodological items, in particular in randomization sequence and allocation concealment [14]. However, these studies did not assess the reporting of intervention features. Others that have assessed the quality of intervention description have not found improvement [17, 18]. For example, a Cochrane review (in its last version published in 2012) exploring the impact of CONSORT Statements on trial reporting found no difference in reporting of intervention between trials published in journals that endorse CONSORT and those that do not [17]. Yu et al. explored in two cohorts (trials published in 2003 and 2013) the reporting of items in CONSORT including the CONSORT extension for Trials Assessing Non-Pharmacological Treatments [18]. While they found overall improvements in reporting in 2013 compared to 2003 they did not do so in items relating to the intervention. Another study reported similar findings when comparing intervention reporting of cardiac rehabilitation over several decades (1975 to 2014) [19]. In sum, these studies show that while there has been improvement in reporting of methodological items in CONSORT this has not extended to intervention descriptions.

It should be remembered that under-reporting is not an issue solely for the intervention [20]. Under-reporting can be found in all documents for clinical trials of both pharmacological and non-pharmacological interventions, from commercial companies and funders’ reports, to systematic reviews and guidelines [21]. However, to our knowledge, our study is the first to explore whether intervention reporting has improved between two 5-year time points separated by the publication of a new reporting guideline.

### Contextualising intervention development

Complex interventions, by the nature of their complex and multicomponent content, are difficult to describe. They are also difficult to develop, and poor description may reflect inadequate attention to the theoretical and modelling phases of their development. Consultation with stakeholders early in the development process is recommended [1] yet very few studies reported that they incorporated patient feedback into their intervention design. This is surprising since the interventions that we focussed on addressed how best to help patients better adhere to their therapy.

#### Implications and perspectives

This study found no overall improvement in the reporting of complex, multicomponent, non-pharmacological treatment interventions. There may be strong evidence that an intervention works but a clinician may be unclear how to use it with their patients as the content of the intervention remains unclear. Researchers may also be unclear how to further develop a promising intervention as they are unsure they have all the details of what it involved.

Awareness raising of the need for reporting adequately interventions should be undertaken early, through education and training starting at university and continuing through professional education and continuous professional development. Moreover, academic institutions could have reporting polices or recommendations for staff undertaking research. Checking that a manuscript adheres to reporting guidelines should not be the last thing an author undertakes prior to submission. Documentation of key aspects of the intervention should be an integral part of the trial development and its analysis. In the case of developing complex interventions, greater attention should be paid early by research teams and research funders to the MRC and other intervention development guidelines. The inclusion of a statistician or other methodologist in a trial team has been demonstrated to improve quality of trial reporting [22].

Publishing groups and journal editors need to endorse reporting guidelines and provide links to initiatives like the EQUATOR guidance that help researchers to formulate research design that is robust and easy to report. Further evaluations need to explore why journals do not recommend CONSORT or other recommendation reporting guidelines, including the more extensive TIDieR Statements. In those that do recommend guidelines it would be useful to explore how well they ensure that authors adhere to them. In a recent survey, it was found that only one of 59 leading pathology journals required authors to submit a guideline-reporting checklist [23]. Journals that implement a policy mandating the submission of a completed reporting guideline checklist for studies have been found to increase compliance and improve the quality of reporting [24].

## Conclusions

This study compared the quality of reporting of complex, multicomponent, non-pharmacological treatment interventions from 2003 to 2008 with 2010 to 2015. It explored reporting in trial papers of interventions to promote adherence to therapy. The study found no overall improvement in reporting.

## Appendix

### Search filters used

#### MEDLINE

1. ((exp patient compliance/ OR (patient adj compliance).tw. OR (patient adj adherence).tw. OR (medication adj compliance).tw. OR (medication adj adherence).tw.) AND ((clinical trial OR random:).mp. OR tu.xs.)) NOT ((qualitative OR retrospective OR mice OR rat OR rats).tw. OR editorial.pt. OR letter.pt. OR comment.pt.) NOT (animals NOT humans).sh.
2. ((random: OR control:).mp. AND (exp patient compliance/ OR patient dropouts/ OR psychotherapy/ OR treatment refusal/ OR patient education/ OR regimen:.tw.) AND (intervention: OR outcome:).tw. AND (medicat:.tw. OR drug therapy/)) NOT ((qualitative OR retrospective OR mice OR rat OR rats).tw. OR editorial.pt. OR letter.pt. OR comment.pt.) NOT (animals NOT humans).sh.

#### CINAHL

1. MH patient compliance+ OR TI “patient compliance” OR AB “patient compliance” OR TI “patient adherence” OR AB “patient adherence” OR TI “medication compliance” OR AB “medication compliance” OR TI “medication adherence” OR AB “medication adherence” NOT PT editorial or PT letter or TI qualitative or AB qualitative or TI retrospective or AB retrospective or TI mice or AB mice or TI rat or AB rat or TI rats or AB rats (limited by Clinical Queries therapy sensitive search filter and date – 2007 to 2012).
2. MH patient compliance OR MH medication compliance OR MH patient dropouts OR MH treatment refusal OR MH patient education OR TI psychotherapy OR AB psychotherapy AND TX ((random* OR control*)) AND TX ((medicat* OR drug therapy)) NOT PT editorial or PT l.

#### EMBASE

(random: or control:).mp. AND (patient compliance or patient dropouts or illness behavior or psychotherapy or treatment refusal or patient education or regimen:).mp. AND (intervention: or outcome: or treatment outcome).mp. AND (medicat: or drug therapy).mp. AND (clinical trial or controlled study or randomized controlled trial).mp.

#### PsycINFO

(((control: or random:).tw. or exp. treatment/) and (adherence or compliance or noncompliance or dropouts or patient education).mp. and (drug therapy or drug or medicat: or treatment or regimen).mp. and (intervention or outcomes or treatment outcomes).mp.) not (qualitative or retrospective or mice or rat or rats).tw.

#### Sociological Abstracts

((patient or treatment or dropouts) AND (clinical trials or control) AND (drugs or medicine or medication)) (Searched using all fields; all(medication) retrieves su(medication adherence).

## Abbreviations

CI: Confidence interval
MRC: Medical Research Council
SD: Standard deviation
TIDieR: Template for Intervention Description and Replication Checklist
